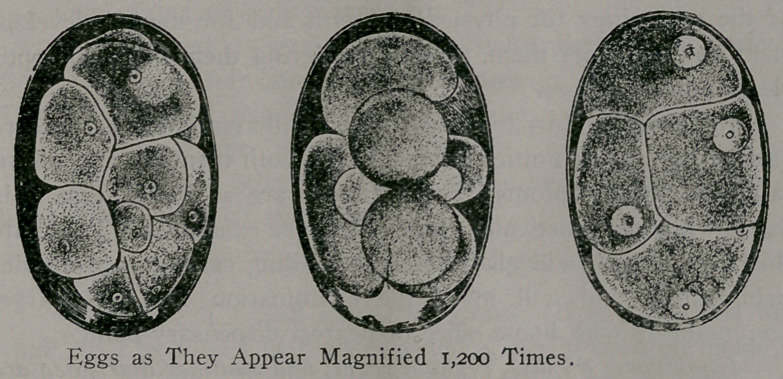# Selections and Abstracts

**Published:** 1911-11

**Authors:** 


### SELECTIONS AND ABSTRACTS.

### THE DUST MENACE AND MUNICIPAL DISEASES*

HOWARD ANDERS, A. M., M. D., PHILADELPHIA.

Among the causes of what may not inappropriately be termed municipal diseases infection, irritation and neurovascular tension play a peculiarly predominant part—most of them an obvious part; some, however, are subtle in their workings. To specify three concrete elements corresponding to and mainly responsible for or contributory to these etiologic processes, there are dust, smoke and unnecessary noise; and the one that looms largest in municipal morbidity and mortality is undoubtedly the widespread prevalence of an irritating as well as infecting dust contamination of our city atmosphere. This dust menace and nuisance is preventable, not only in the streets, as is now demonstrated in a few large cities, but likewise in our living and working places, in houses, cars, stores, factories, shops, offices, stations, theatres and churches; in fact, there is a vicious circle here in that street

*Read before the American Climatological Association, Montreal, Canada, June 14, 1911, and reprinted from the Journal of the American Medical Association, Nov. 4th, 1911.

dust is blown, swept and tracked into habitations, to be swept back into the streets again. Much house-dust pollution would be avoided if the original street-dust was removed or allayed. Says Dr. George A. Soper of New York: "There is something incongruous about a board of health conducting a crusade against smoke and noise and at the same time allowing the streets to be filthy with dust and dirt."

In biographic and occasional references to Benjamin Franklin, it is the usual thing to have pointed out to us his many talents and rare common sense as well as his courageous patriotism and eminent public service. It is, however, perhaps as entertaining and interesting as it is instructive and exemplary to read in the "Autobiography" of his study and efforts relative to dirty, dusty streets in Philadelphia and London, as far back as a century and a half ago. Here as elsewhere does he reveal the master mind in originality and acuity of perception, versatility of faculty, and prompt practicality of action. The dust evil was a seriously annoying problem to him as a citizen if not as a sanitarian; to him dust was at least noisomely offensive if not infective. He writes of his proposal to Dr. Fothergill of London—whom he commends as a "great promoter of useful projects"—that "for the more effectual cleaning and keeping clean of the streets of London and Westminster," contracts be let to have the dust swept up in dry seasons, especially before the shops and windows of the houses are open, and that the gathered heaps of refuse be carried away in close-covered carts. Could Franklin view the methods and results in London, Toronto, Berlin and some other cities to-day, his only regret might be that it had taken so long to inaugurate the improvement; and that so many cities still drag along, apparently contented and unable to cope with a distressing, dust-polluted atmosphede, even worse than in his own day, by reason of the aggravations of modern speed traffic. He concludes:

"Some may think these trifling matters not worth minding or relating; but when they consider that though dust blown into the eyes of a single person, or into a single shop on a windy day, is but of small importance, yet the great number of the instances in a populous city and its frequent repetitions give it weight and consequence, perhaps they will not censure very severely those who bestow some attention to affairs of ths seemngly low nature."

Now that for about seven years street-dust as an element in the propagation of disease, especially in large cities, has received such marked and spreading recognition, the problem of street as well as house cleanliness is a live and urgent one. Modern civilization, being mainly materialistic and commercialistic, is responsible for the creation of a multifarious, mixed, material refuse: street-dust is such a pernicious and persistent menace to the public health because it bodies itself forth as veritable synthetic pulverized poison composed of various more or less palpable particles of mechanical, chemical and bacteriologic substances, both irritating and infecting when inhaled.

As long ago as 1870, Tichborne and Tyndall showed that most of the fine dust particles suspended in city atmosphere are composed of organic matter, and bacteriology has since shown that the invisible part of visible dust, that is, its germ content, is the dangerous element that calls for a drastic and determined movement for the suppression and removal of the menace everywhere. This applies mainly to cities.

Country road dust is composed principally of ground-up rock and is a comparatively clean dirt, irritating to the mucous membranes but relatively innocuous as to pathogenic organisms. Besides, the task of dust-allaying must obviously for some time to come remain a municipal one, excepting in those suburban and more populous, much-trafficked and prosperous outlying communities where even now many miles of macadamized and Telford roads are oiled, tarred, or sprinkled with water.

Apart, however, from the modicum of mineral dust arising from the wear and tear of street pavements by horse, wagon and railway-car traffic, municipal dust represents the accumulation of debris of various kinds, animal and vegetable, such as dried sputum of human origin, horse and dog droppings and slobberings, dead and disintegrated insects, scattered garbage and house sweepings, loaded vehicle losses, smoke and soot—in short, mainly an organic combination which in warm, wet weather is seething with rottenness; and in dry weather is blown into our houses, noses and clothing by gusty winds, or sucked up as noxious, stifling clouds by motor cars while they speed and trolley cars while they are running or braking for a stop.

To the average pedestrian, dust is hardly more than an annoyance; hence arises the large opportunity and high duty of dust; to show that it is not simply an irritant to the eyes, nose and throat, but a serious carrier of infection by inhalation and swallowing; that the pus-producing germs, so abundant in such dust, may and do infect and produce purulent conjunctivitis, boils and carbuncles, and the graver, purulent catarrhs of the nasal, faucial and bronchial tissues.

That most of the communicable diseases of the respiratory tract are produced and transmitted by dust is doubtless true. For years we have had before us the deadly relation of the dust evil to tuberculosis. Were that the only danger, surely the awful prevalence and mortality-rate of the "great white plague" would alone justify the fight for street cleanliness and a declaration of independence against dust distributers. But when one adds to this the host of other dust-borne diseases, catarrhal, tonsillar, bronchial, influenzal, pneumonic, rheumatic, cutaneous, the need for efficient public service in remedying and removing the evil becomes intensely imperative and the demand inexorable for right results.

Epidemics of purulent catarrh, tonsillitis of severe type, influenza, bronchitis, sore eyes, boils, have frequently and commonly been associated with, or have closely followed, prolonged meteorologic periods of dry, gusty weather, when it was too cold to sprinkle the dirty streets properly, or when the city authorities have been too careless in beginning the cleaning and sprinkling of streets early in the season.

Dust particles are necessary nuclei for the formation of fogs (as experimentally shown by Carl Barus) and the suspension of atmospheric moisture: and thus a two-fold unhygienic relation of dust should be brought to mind. In the first place, an excessive amount of municipal dust, attracting to itself a corresponding amount of moisture, actually renders the sensible humidity of the city air more unbearable and debilitating generally. Secondly, conceding "that pathogenic organisms are adherent to particles of dust of various kinds, and that their retention of virulence depends on the amount of hygroscopic moisture with which they are associated," as Harrington points out, the more they are protected by such hygroscopic particles, the longer they will sustain the viability to which such moisture is essential, especially in warmer temperatures.

It is a needful thing in public health teaching to dispel the notion—the obsession it has almost become—as to "catching cold." Truly, most of the so-called "colds" are dust infections gotten in places where people congregate and traffic circulates freely, as in street cars, theatres, department stores, moving picture shows, halls, etc. As Woods Hutchinson has aptly put it, "colds might rather be called "fouls." Human agency is principally at fault in polluting the air with disease-bearing dust, and human agency and energy must be associated and directed to arrest, subdue and prevent its production and propagation. The so-called air-borne diseases of great cities are really synonymous with dust-borne. Instead of bad air in the vague sense, let us speak of dusty air in the definite sense; instead of the Italian malaria, the Greek derivative *konisaria* (*konis* dust; *aer,* air.)

One of the most significant discoveries of recent times in clinical medicine is the not infrequent causative relation of follicular tonsillitis to the early development of rheumatic, endocarditic and pericarditic complications and sequellae. On first thought it may seem like a startling and incredible statement to make that dust may at times infect the tonsils, and through these portals cause the most serious and malignant involvements of joint and heart structures; and yet sufficient medical literature and my careful study of several cases demonstrate clearly that the links of the pathologic process do connect.

Dust, then, is not by any means or in the main a severe nuisance and source of public discomfort, but perforce a menace and direct danger of deadly potentiality.

Further still, as a habitually inhaled mechanical and sometimes chemical irritant alone, dust predisposes the upper air passages to later if not occasional associated bacterial implantation because of the production of a catarrhal soil of ready receptivity and decreased local vital resistance; the germs are not only invited, but embraced and encouraged to stay, multiply and penetrate to the innermost parts of a body which is at the same time somewhat generally debilitated and vulnerable. Here again comes forth a vicious circle. The more dusty the air in our cities, and the more consequent catarrhal irritation, the more sputum, and the more spitting promiscuously in public; and the more people spit the more infectious and dangerous the dust becomes.

The street-cleaning problem cannot be disassociated from the problem of the enforcement of anti-spitting laws. People with nasopharyngeal and bronchial catarrhs must expectorate somewhere; and for many years to come they will do so on our highways. It is practically difficult and a great hindrance to progress in this matter of preventing or lessening expectoration on our streets as long as inadequate and inefficient means and methods of street-cleanng and dust-allaying fail to control or suppress the dust evil.

As the anti-dust campaign, in Philadelphia at least, got its first impetus from the point of view of the prevention of tuberculosis, a special reference to the relation of dust to this widespread disease may be made with propriety. First, the constant inhalation of all kinds of irritating dust makes the invasion of the tubercle bacillus easy. This is well known not only as regards occupational, mineral and organic dust, but particularly constant to municipal street-dust and house-dust of ordinarily constant prevalence. General and local predisposition are coupled in that the environment conduces to the former equally with the latter. Secondly, although less commonly, direct infection with the tubercle bacillus from street-dust may occur from recently expectorated tuberculous sputum that has dried rapidly under a strong wind on a cloudy day; but habitual infectons from house-dust are, of course, the usual modes. Thirdly, mixed infections with the common dust germs of pus formation, the staphylococci and streptococci, may occur as well as with bacilli of pneumonia and influenza. It is to these that we must lay the great burden of the aggravations, complications and fatal terminations of our tuberculous patients. A simple, curable, early case becomes difficult or impossible to jugulate because of a mixed dust infection, and consequent extensive and progressive caseation and cavitation. How many hopeful, more or less curable, tuberculous patients have run a rapidly fatal course because of a daily inhalation of a foul street-dust will never be known.

Says Jacob A. Riis: "All things come to those who wait —and fight for them." The history of the agitation against the dust menace in Philadelphia, and the results obtained there to-day, while yet far from satisfactoryy, show that educative repetition of the facts and remedies at hand will bring about substantial improvement, and often sooner than expected, a quite rapid progress to still better conditions. In 1903, while president of the Pennsylvania Society for the Prevention of Tuberculosis, I inaugurated a campagn of publicity against the dust evil, particularly as related to the dirty streets and cars. For two years more the attention of the public was called to the dangers of dust, and an endeavor made to have the proper municipal authorities adopt and practice modern means and methods of street-cleaning and dust allaying. The Philadelphia Rapid Transit Co. (which operates all the trolley-cars in the city) responded to the agitation by abolishing the foul, dusty plush cushions. The city officials were inclined to resent interference with their inertia and indifference, but the criticisms began to tell to the extent that sporadic attempts at better cleaning and sprinkling of streets were made at irregular intervals.

In January, 1909, as chairman of the legislative committee of the society, I took up the work with renewed vigor. Attention was again called to the disease-predisposing and infecting dangers attaching to the actually filthy conditions of the streets, and to the stupid, ill-timed, incompetent ways of attempting to clean them; a dry dabbling method of sweeping, in spots at that, with no preliminary thorough sprinkling or flushing of the asphalt pavement, and this done in daytime in the most frequented streets, causing clouds of dust to be raised to annoy and to endanger thousands of pedestrians. Also the heaps of sweepings, even when moist, were allowed to remain for hours and become dried by the sun and the wind, and usually scattered about again by air-gusts and automobiles. Public opinion began to recognize that such slipshod, lackadaisical inefficiency was due to an inexcusable irresponsibility, if not a shameless cupidity on the part of officials and contractors who could thus disregard public health.

Simultaneously I issued circular letters of queries to the mayors of some twenty-five cities of America and Europe as to the methods and results of cleaning, dust-reducing, and keeping clean the streets of their respective jurisdictions. The replies from the principal cities were tabulated and afforded a basis for a comparative study of the situation, and the results were published in the spring months of 1910 in a series of twelve articles in a few of the newspapers of Philadelphia, mostly in the *Public Ledger.* The cities reporting were twenty-one, including Philadelphia, and stated the requirements of ordinances, what was actually done and how, and with what degree of satisfaction and sanitary safety, according to official opinion. To enumerate the cities alphabetically, there were Baltimore, Buffalo, Birmingham (England), Berlin (Germany), Budapest (Hungary), Cleveland, Denver, Detroit, Dublin (Ireland), Leipsic (Germany), London (England), Minneapolis, New Orleans, New York, Paris (France), St. Louis, Stuttgart (Germany), Toronto (Canada), Vienna (Austria), and Washington, D. C. I was able to supplement these reports by personal observation in some of these cities and in Munich, Germany. To summarize:

1. In a majority of the cities where results were satisfactory, the principal cleaning was done at night, or completed before 6 o'clock in the morning.

2. Thorough sprinkling preceded sweeping. Sprinkling was also done two to four times daily in dry (non-freezing) weather to lay prevalent dust.

3. Flushing wagons or flushing from curb to curb with hose is the method used in preference to sprinkling and machine sweeping in the best cleaned cities. Indeed, sprinkling is seldom done where the streets are thus virtually washed two or three times weekly.

4. Hand sweeping by blockmen in day-time i salways preceded by wetting with a hand sprinkler.

5. Piles of dirt are immediately removed while wet or damp in covered wagons.

6. Last, but by no means least—and the method which in my judgment is destined before long to supercede all others in street cleaning—is the dustless operation of dust and dirt removal by automobile vacuum street-cleaners, on the principle of vacuum house cleaning which is now rapidly taking the place of the old dirty, dust-raising broom and feather-duster. Recent tests of these street machines have developed the fact that in one hour as much surface can be actually cleaned as was imperfectly gone over with dusty accompaniments by horse-drawn sweepers in six hours.

It was pointed out by contrast that in Philadelphia and some other cities it was adding insult to injury to attempt to clean the street by methods that aggravated instead of mitigated the dust evil, discomfort and danger. Sprinkling before sweeping was specified in contract but never carried out. Hence the criticism was urged that not only better means and methods needed to be adopted, but more systematic and adequate inspection and supervision of the work done and the methods specified, and a more efficient management generally by the city street-cleaning officials, whether the work was done by the government or by contractors. Otherwise, it was futile and farcical to expect a lessening of the menace of dust. The blame for dirty streets should not be placed on the day laborer who may shirk his job. He is only following an example higher up.

The people were appealed to in these articles, and a public protest sought. If they were insistent and persistent in their demands for clean streets, the city government would be equally insistent in its demands on the contractors. The public was reminded that streets are the ventilating flues of city, and as a result house ventilation depends much on the purity and quality of street air; whereas, dirty, dusty streets become above-ground air sewers instead of pure air channels.

It soon became evident that a dust-infected people was becoming discontented, and that a city healthful was of greater importance than a city beautiful: in fact, that civic cleanliness made for civic beauty and art. Tax-payers began to realize that they were not receiving an equity of public sanitary safety.

Education has been telling, and the street-cleaning authorities have been seeing a great light in Philadelphia. The methods have improved; the work is more efficiently done; flushing wagons have recently been introduced with effectiveness, but of course too few as yet, and capable of attending to no more than a half dozen of the busy down-town streets. A steady, strong fan-shaped coluumn of water is driven laterally by the pressure of a gas-engine on the rear of the wagon so that all debris is flushed into the gutters from the middle and sides of the streets; the wagons are horse-drawn, passing in pairs, one a little in advance of the other; the streams of water being directed in opposite directions.

It is a hopeful sign of the awakening of the public to demands for civic cleanliness, that officialdom is beginning to respond in a readier performance of its duty and responsibility in safeguarding the public health and incidentally safeguarding respect for law, order and government. To avoid an insurrection of a citizenship, there must needs be a resurrection of statesmanship.

---

### TIFT COUNTY AND GEORGIA STATE BOARD OF HEALTH CAMPAIGN AGAINST HOOK-WORM DISEASE.*

Most prevalent in Georgia. It has been found in practically every county in the State.

### The Signs of the Disease.

1. Ground itch is the initial symptom; 2nd, Indigestion; 3rd, Pale skin; 4th, stunted growth; 5th, Lack of energy; 6th, Inattention to school work and inability to progress satisfactorily; 7th,

Dull, listless expression with glistening eyes; 8th, Something the matter, BUT DON'T KNOW WHAT.

Of course all of these signs are not found in every case. The worst cases show them together with swelling of the feet and so-called dropsy. Many have only the indigestion, or the feeling that somethng is wrong with them, they know not what. Hookworm disease is common with ALL classes of people and

Hookworm's Natural Size.

all races of people. The young contract it more often than the old, because in Georgia nearly every child goes barefooted. After putting on shoes, and freeing yourself from ground itch, in the course of from EIGHT TO FIFTEEN YEARS the worm will die.

Eggs as They Appear Magnified 1,200 Times.

These worms attach themselves to the intestine, and sap one's blood. The disease is diagnosed by finding the eggs in the passages from the bowels. The eggs can be seen only under the microscope, but the worms can be seen with the naked eye, each being about 1-2 of an inch long, and about the size of a number one sewing thread.

A. Hookworm disease is the condition caused by the worm being attached to the intestine, and sapping one's blood, also injecting a poison into the remaining. It is more common in the warm climates because the eggs hatch out better here.

B. The eggs develop into baby hookworms which penetrate the feet of the barefooted children. They are taken into the circulation, carried to the heart, and pumped to the lungs, there they burrow through the tissue of the lung often causing coughs, and they then crawl up the bronchial tubes into the throat where they are either swallowed or coughed up.

After they have gone through this process, they are developed sufficiently to attach themselves to the intestine.

C. These worms do not multiply in the intestine, but lay eggs there. These eggs pass out into the soil where they are hatched.

The improper disposition of night soil or human excrement is the cause of the spread of the diseases. *One person on a farm having the disease, may, by careless habits of soil pollution, infect a whole farm.*

E. The effect of the disease on a person is to deprive them of the necessities for physical strength and for mental development and to dwarf them, thereby rendering them more susceptible to other diseases.

F. It is our duty to see that every child entrusted to our care has the best opportunities of developing both mentally and physically and the symptoms of this disease are so varied that it is worth while for practically every child to be examined. The State Board of Health will gladly furnish mailing cases to anyone desiring them, and will make the examination free of charge. Communicate with home office or nearest dispensary point.

*Hookworm disease, typhoid fever, and summer diarrhoea are often caused from the lack of proper sanitary surface closet around the homes, school houses and churches.*

G. To prevent the spread of this disease those who are infected should be *treated*, and each home, each school house, each church should be provided with a *sanitary surface privy*, so as to prevent the spread of hookworm disease, typhoid fever, and summer diarrhoeas. Wear shoes on damp, warm days. Build and properly care for a Sanitary Surface Privy.

### Sanitary Surface Privy.

It is impossible to overestimate the value to health of the proper building, use, and care of the closet. By properly building the same, flies can be screened from the contents of the vaults, thereby preventing soil pollution and the spread of hookworm disease, with its fearful results; also the protection of the water supply from contamination. Privacy is inbred and we are most apt to form habits, the value of which to health, not to speak of character, will be great.

### Bill of Material Needed.

36 running feet 2 x 4.
100 running feet 2 x 3.
16 running feet 4 x ⅞.
2 pieces matched boards 4 ft. long by 9 in. wide, or 1 piece 4 ft. long by 18 in. wide.
250 ft. ⅞ in. boards.
250 ft. strips or battens.
One spring or pulley for door.
8 ft. screen, 15-mesh copper or galvanized, 12 in. wide.
2 hinges, 6-in strap, for front door.
2 hinges, 6-in., "T," for vault door.
4 hinges, 3-in. "butts," for covers.

Cost.—From $6.00 to $12.00, depending on local price of lumber and grade of stock used.

### Explanation of Cuts.

Figures 1, 2, 3, and 4 are flat views. It is preferable that vault door as shown in Figures 2 and 4 should open on inside at point as shown in Figure 3 marked seat, and not in back.

Figures 5, 6, and 7 show all sides of building, together with vault, during construction. The door in back as shown in Fig. 6 can be swung in front of vault, Figure 7. This is preferable.

Figures 8, 9, and 10 show construction of door, screen holes, and seat.

Frame.—Heavier framing can be used, and is, of course, preferable; 4 x 4 could be used in place of 2 x 4, and 2 x 4 instead of 2 x 3. We will refer to the lighter material.

Cut 7 pieces 2 x 4 ft. long. Place 3 of them on level ground at right distance for girders. (G, Fig. 5.) Toe-nail (Fig. 11) firmly the remaining four joists (J. Fig. 5.) Care should be taken to have the corners of this frame square. Raise same about 2 in. above ground by placing brick or flat stone under it. Carefully level it.

FLOOR.—Nail on floor boards, making a square platform 4 ft. square.

POSTS.—Take 4 pieces 2 x 3 and saw one end of each perfectly square. The square end should fit well on the floor. Place 2 of these against a straight piece of board (Fig. 12) so that the ends are 4 ft. apart and the pieces square to the board. Now measure upon the outside edge of one piece 8 ft. 3 in. and on the inside edge of the other 5 ft. 10 in. Place a straight board across these marks and draw a slanting line across the 2 x 3 (Fig. 12.) Saw on these lnes and you have two posts. Make other two same way.

BRACES.—Cut 5 pieces 3 ft. 8 in. long and 3 pieces 3 ft. 6 in. long (x and y, Fig. 6.) Be careful that the brace in back is right height to make header for door, if you prefer door in back.

RAFTERS.—Nail a piece of 2 x 3 lightly across side of posts, and even (flush) with the slanting ends. Mark the piece on the inside, knock it off, saw and fit in. (R, Fig. 6.)

Nail other 2 braces (x) between the rafters, turned just right to carry the roof boards.

WALLS.—Select eight pieces of board, mark out openings for the screens (Fig. 9), be careful that the one for the vault is far enough down. (See Fig. 2.) Take the planks, mark them to fit as shown in Fig. 5. Be sure and leave the opening for vault door, if the open back style in used.

ROOF.—Cut the roof boards 5 ft. 9 in. long. They should hang over 6 in. in front and back, and 4 or 5 in. at the sides. Nail them in place as shown in Figs. 5 and 6. See that there are no cracks for flies to crawl through. If so, cover them.

BATTEN OR STRIPS.—If matched boards are used no battens or strips will be needed on walls, but to make the roof water tight, they must be used over the cracks, as shown in Figs. 4 and 6.

If matched boards are not used, they must be used over the whole house.

SEAT.—Frame the seat as shown in Figs. 5 and 6 and nail on the front as shown in Fig. 7. It is better to have same braced with 2 x 3s and the door made to open in the front of the vault, care being taken to make it sufficiently large for the easy removal of the receptacle.

The seat is made of two pieces of board 4 ft. long and 9 in. wide, or 1 piece board, 4 ft. long and 18 in. wide, matched boards to be used. See Fig. 7 for construction of the hole.

Fig. 12 shows an easy method of marking off the hole by means of a cardboad. Nail is driven through one hole, and 4 in. from it a pencil is inserted into the other. Round the front of the seat as shown in Fig. 15. All openings left back of seat should be completely closed. Nail a strip (V) at the back to carry hinges for the covers.

Make the covers and nail the strips (W) in place at the sides.

DOORS.—Make the door for the front as shown in Fig. 8, and for the vault, if the open back method is used, as shown in Fig. 6, but preferably the door for the vault should open in front, as shown in Fig. 7.

SCREENS.—Every opening should be screened. Make a frame Fig. 16, to fit tight over the door, tack wire screening (copper is best. but galvanized iron is good), over each opening and cover the edges with strips as shown in Fig. 17.

Never leave the door or vault open. Put a string or pulley on the door so it cannot be left open carelessly.

VAULT BUCKET.—The best is a large coal scuttle, but any can or tub may be used; care being taken that the top should be only 2 or 3 in. from the hole.

These cans should be cleaned at least once a week, or more often if necessary. The fecal matter should be burned or buried at least 200 feet from the well and at a place slanting from the house and well.

A little dirt or disinfectant can be sprinkled into the vault occasionally, and will greatly aid in keeping down bad odors.

### ARMY MEDICAL CORPS EXAMINATIONS.

The Surgeon General of the Army announces that preliminary examinations for the appointment of first lieutenants in the Army Medical Corps will be held on January 15, 1912, at points to be hereafter designated.

Full information concerning these examinations can be procured upon application to the "Surgeon General, U. S. Army, Washington, D. C." The essential requirements to securing an invitation are that the applicant shall be a citizen of the United States, shall be between 22 and 30 years of age, a graduate of a medical school legally authorized to confer the degree of doctor of medicine, shall be of good moral character and habits, and shall have had at least one year's hospital training as an interne, after graduation. The examinations will be held concurrently throughout the country at points where boards can be convened. Due consideration will be given to localities from which applications are received, in order to lessen the traveling expenses as much as possible.

The examination in subjects of general education (mathematics, geography, history, general literature, and Latin) may be omitted in case of applicants holding diplomas from reputable literary or scientific colleges, normal schools or high schools, or graduates of medical schools which require an entrance examination satisfactory to the faculty of the Army Medical School.

In order to perfect all necessary arrangements for the examination, applications must be complete and in possession of the Adjutant General at least three weeks before the date of examination. Early attention is therefore enjoined upon all intending applicants. There are at present sixty-four vacancies in the Medical Corps of the Army.

---

Vaginal hysterectomy is more dangerous than abdominal hysterectomy when the uterus is adherent.

The "safe-triangle or "interpleural space," for exposing the heart, is at the left edge of the sternum behind the three lower costo-cartilaginous attachments.

—*American Journal of Surgery.*

### PROCEEDINGS OF THE AMERICAN PROCTOLOGIC SOCIETY AT LOS ANGELES, CAL., JUNE 26 and 27, 1911.

### EXTRACTS FROM THE REPORT OF PROCTOLOGIC LITERATURE FROM MARCH, 1910 TO MARCH, 1911.

By Samuel T. Earle, M. D., of Baltimore, Md.

*(Continued from Last Issue.)*

*A Paper: Intestinal Stricture Following Ileo-Rectostomy. Report of a Case Was Read.—By Frank C. Yeomans, M. D., of New York City, N. Y.*

J. X., a man 46 years of age, was always strong and well, but suffered from severe constipation of many years' standing. In October, 1909, an anterior sigmoidopexy was proposed for "prolapse of the sigmoid." Temporary relief followed, but three months later "peritonitis" developed. The same surgeon operated again, freed numerous adhesions, divided the ileum just proximal to the colon, closed the abnormal end and implanted the oral end of the ileum into the rectum. Relief of the constipation was prompt, but when he first consulted Dr. Yeomans, in July, 1910, it had returned in an obstinate form with all the symptoms of a marked auto-toxemia superadded.

The proctoscope passed easily, but no opening could be discovered in the rectum or the sigmoid. An excellent radiograph, by Dr. L. G. Cole, proved the colon and sigmoid to be unobstructed.

Concluding that the feces, following the path of least resistance, were accumulating in the colon, Dr. Yeomans did an appendicostomy at the N. Y. Polyclinic Hospital, December 16, 1910. Irrigations through the appendix relieved all symptoms for ten weeks. Constipation and toxemia then returned, however, and he performed an exploratory laparotomy March 14th, 1911. The ileum ran down into the left side of the pelvis and was lost in a mass of dense adhesions. A broad lateral anastomosis was made between the ileum, just above the adhesions and the sigmoid. The patient reacted well from the operation, but developed a double pneumonia 18 hours later, to which he succumbed on the fifth day. The urine was suppressed the last 24 hours of his life. The bowels moved on the second day, and, thereafter, three or four times daily. At the autopsy no peritonitis was found. The specimen removed, consisting of ileum, sigmoid, and rectum intact, showed perfect unison of the recent lateral ileosigmoidostomy. The remarkable feature of the old end-to-side ileo-recostomy was that the opening was so constricted that it would scarcely admit a 16 F. catheter and physiologically amounted to a stricture.

The noteworthy features of this case were:

1. Reverse peristalsis of the colon, evidenced by the large quantity of feces expelled by the irrigations through the appendicostomy.

2. The radiograph was valuable in demonstrating a patent sigmoid and colon, thereby proving that the obstruction was in the small intestine.

3. Failure of the proctoscope to reveal the site of the opening does not discredit the diagnostic value of that instrument but shows the extreme degree of contraction of the opening.

4. The many actions of the bowel signify clearly that the physiological function would have been permanently restored had the patient survived the pneumonia. The practical lesson derived from a study of the case is that lateral anaestomosis is superior to end-to-side union, especially in the presence of inflammation.

---

*Syphilis of the Ano-Rectal Region.—By Lewis H. Adler, Jr., of Philadelphia, Pa.*

The author related the history of two cases of syphilis in which no outward visible effects of the patient's grave condition existed, except about the anus. In both instances, the anus was surrounded by syphilitic condylomata; the parts were bathed in a fetid sero-purulent discharge and the patients' mouths were affected with mucous patches. In one case the patient was markedly improved by the use of salvarsan and the other one improved under the ordinary mercurical treatment, but disappeared from observation before a cure could be effected.

The writer then took up the consideration of the usual manifestations of the disease as affecting the localities under consideration, stating that the primary lesion, always a chancre,—occurs about the anal region much more frequently than is usually supposed. That chancre of the rectum proper, in this country, is a very rare occurrence. Where sodomy and other unnatural vices are practiced, infection may, and possibly does, occur with greater frequency. That females are oftener affected than males and while the occurrence of the initial lesion about the anus or within the rectum of men, is almost positive evidence of the practtce of sodomy; in women, the possibility should be remembered of the infection of these parts arising through contact with the male organ, or from the vaginal discharges.

That the diagnosis of all doubtful cases of syphilis can now be definitely determined when the patient's blood shows a positive Wasserman reaction and by finding the presence of *spirocheta pallida.*

Attention was called to the fact that cases of ano-rectal syphilis develop the usual smyptoms of the disease as when it affects other parts of the body. and, next to the mouth and throat, the anus is the most frequent site for mucous patches.

Attention was called to the hereditary or congenital form of the disease; and, among the tertiary lesions, the following principal varieties were enumerated:—Gummata, destructive ulceration, stricture, ano-rectal syphiloma, and proliferating proctitis.

The article concluded with a brief consideration of the treatment of the disease in which attention was directed to the necessity of care being exercised in looking afer the hygiene in all its phases: that the constitutional treatment of the disease should not be commenced until a positive diagnosis is established; that as no on eform of mercury, or any one of the various methods of its administrations may be employed successfully in all cases, the individual requirements of each person should be the guide.

Ehrlich's remedy—salvarsan—had in several instances been employed with excellent results, but the author would not depend upon its employment alone, believing that mercury should supplement its use.

In the use of salvarsan, it was advised that no one treat patients with it, except those specially trained in its preparation and administration.

---

*Foreign Bodies in the Rectum.—By T. L. Hazzard, M. D., of Pittsburgh, Pa.*

The paper consisted mostly of a recital of four recent cases of foreign bodies in the rectum. Two were in children, in which the substances were accidentally swallowed, and the others were adults who introduced the bodies directly into the rectum through some perversity.

Case 1. Baby girl, two years old. Referred for dysentery of three months' duration. The chief symptoms being bloody stools, mucus and tenesmus. No digita l or other examination had previously been made. Examination with the little finger showed the presence of something lying across the bowel, low down. A guarded pair of scissors was introduced and this body was easily cut in half and removed. It proved to be a match, or at least, two-thirds of one. Although the ends of this match were firmly fixed in the sides of the intestine, no abscess followed. Recovery was rapid and uneventful.

Case 2. Boy, a little older than the first case. The symptoms, conditions and procedure were the same as the preceding case, but the foreign body was a bone from a frog's leg.

These cases show the necessity for rectal examinations. In one case a bacterial microscopical test had been made, but was rather misleading than otherwise.

Case 3. Self-introduction into the rectum of a prescription bottle, a "Baltimore oval," 3 oz. The mouth was upward. After considerable trouble, it was removed by means of a blunt hook. It had been in the bowel for three days.. No anaesthetic necessary. The case progressed without any untoward incident. He gave no reason for his action, and no questions were asked, as he would not have told the truth.

Case 4. Adult, aged 45. Had been a cow-puncher. At present has no occupation. Came to Alleghany General Hospital

Examination showed the presence of a very thin beer glass, 2 inches wide, at the top, and 3 1-2 inches tall. Spinchters contracted. No bleeding and but little discomfort. In attempting to remove it, it was broken. After it was extracted there was considerable bleeding from the rectum. He developed pelvic peritonitis and a rather large tumor developed in the left iliac region. This passed away and he was discharged in about three weeks, not altogether well of the pelvic pains.

General treatment in all cases was rest in bed, with frequent washing of the bowel with a 1% solution of creoline and normal salt.

---

*The Limitations of the Use and the Methods of Employing Local Anaesthesia in Rectal Surgery.—By Lewis H. Adler, Jr., M. D., of Philadelphia, Pa.*

The author, quoting from a recent article of a distinguished proctologist states:—"Patients seriously object to a general anaesthetic and because of this and the fact that most minor anorectal operations can be painlessly performed under local anaesthesia induced by sterile water, or a one-eighth of one per cent. eucaine solution, I have discarded general narcosis in about eighty per cent. of my rectal operations."

In taking exception to this general statement he questions the wisdom of sending it broadcast and advocating a methods which, in the hands of one not particularly skilled in rectal work, would, in his opinion, only lead to disaster.

He calls attention to the water logging of the tissues, when sufficient anaesthetic be used, whether cocaine, eucaine, sterile water, or other agents, and to the subsequent retarding of the recovery of the patient and the danger of hemorrhage from allowing patients to be about on their feet, citing a case which proves conclusively the force of his arguments.

The author claimed a thorough understanding of the underlying conditions can rarely be made without the aid of general anaesthesia. The latter, when administered by a competent anaesthetizer is not attended with any more danger or risk than the indiscriminate employment of local anaesthesia.

He calls attention to the fact that it is essential to remove the anaesthetic when the spinchter is divulsed, as deep inspiration thus induced would cause too much of the drug to be inhaled suddenly, and might cause alarming or fatal results.

Rectal diseases, which may be treated under local anaesthesia, he considers under two divisions:—(1) those admitting of office treatment; (2) those requiring treatment at home or in a hospital.

In the opinion of the author, external piles or other excrescences around the anal region, some fissures-in-ano, and abscesses (of not too large an extent), are the only affections coming within the range of operations which can with propriety be performed in the office under local anaesthesia. He warns the operator that trivial fistula, often have diverticulae and are not readily discoverable except under general anaesthesia.

Under the second heading he speaks of internal colostomy and internal hemorrhoids and warns the operator that the temperament of the patient must always be taken into account. Highly nervous patients will not stand manipulation of the intestines and the abdominal muscles are apt to be rigid.

The author mentions the different drugs used in local anaesthesia, the vibratory method of Hirschman, the methods used in getting the parts anaesthetized and the after treatment.

The trend of the article is not to throw cold water on the valuable procedure of local anaesthesia, but to insist that the cases must be suitable and in the hands of men of experience.

## Figures and Tables

**Figure f1:**
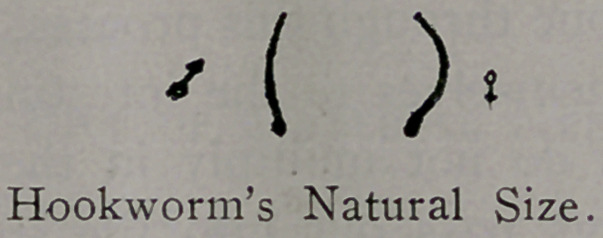


**Figure f2:**